# Supplementary material for: ADCK2 Knockdown Affects the Migration of Melanoma Cells via MYL6
Source: Cancers (Basel). 2022 Feb 20;14(4):1071. doi: 10.3390/cancers14041071 (PMC8869929; doi:10.3390/cancers14041071)
Supplement: Supplementary file 1 [file cancers-14-01071-s001.zip › cancers-1546617-supplementary-original image.pptx]

## Slide 1
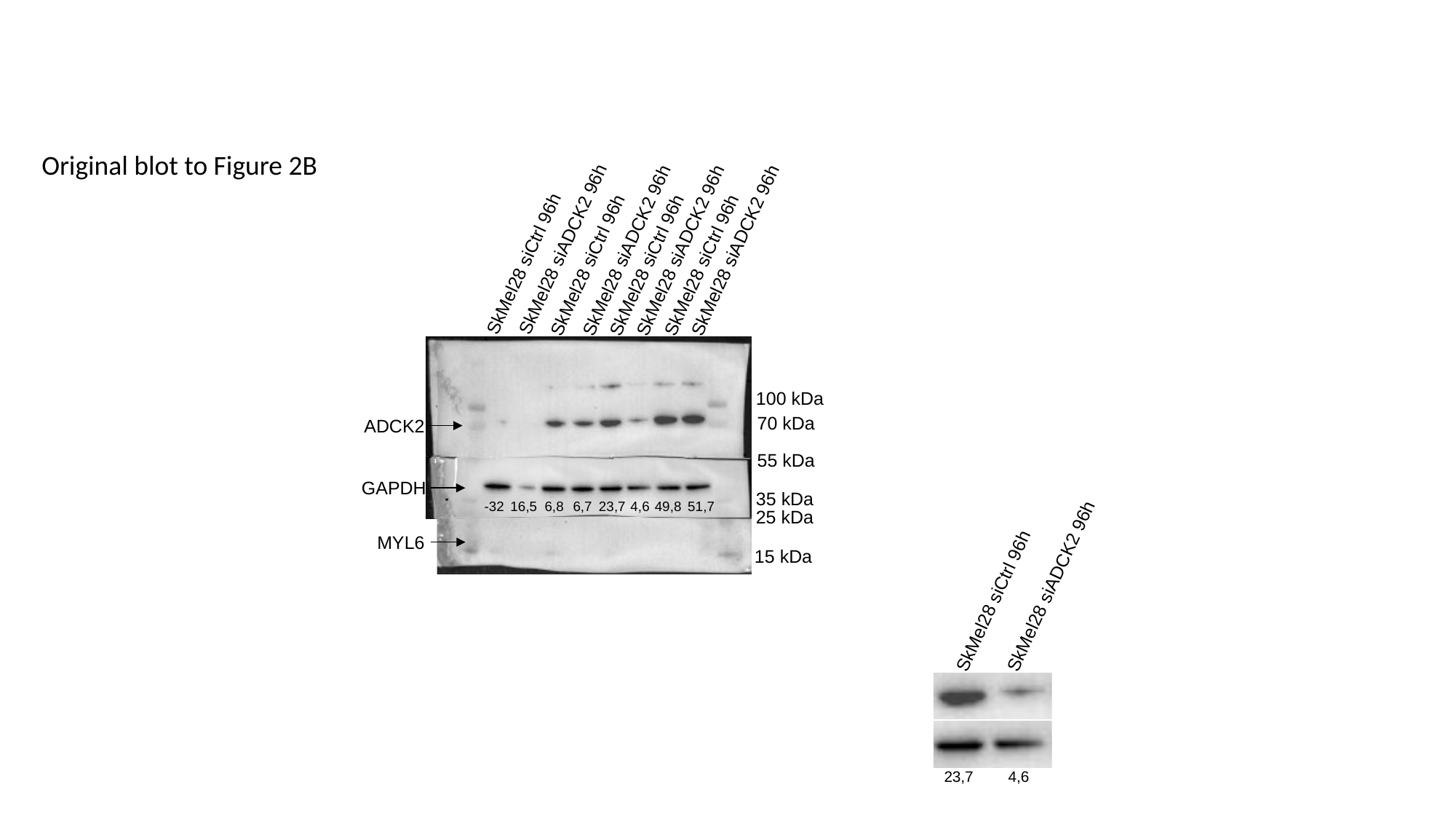

SkMel28 siADCK2 96h
SkMel28 siADCK2 96h
SkMel28 siADCK2 96h
SkMel28 siCtrl 96h
SkMel28 siCtrl 96h
SkMel28 siCtrl 96h
SkMel28 siADCK2 96h
SkMel28 siCtrl 96h
Original blot to Figure 2B
100 kDa
70 kDa
ADCK2
55 kDa
GAPDH
SkMel28 siADCK2 96h
SkMel28 siCtrl 96h
4,6
23,7
35 kDa
-32
16,5
6,8
6,7
23,7
4,6
49,8
51,7
25 kDa
15 kDa
MYL6

## Slide 2
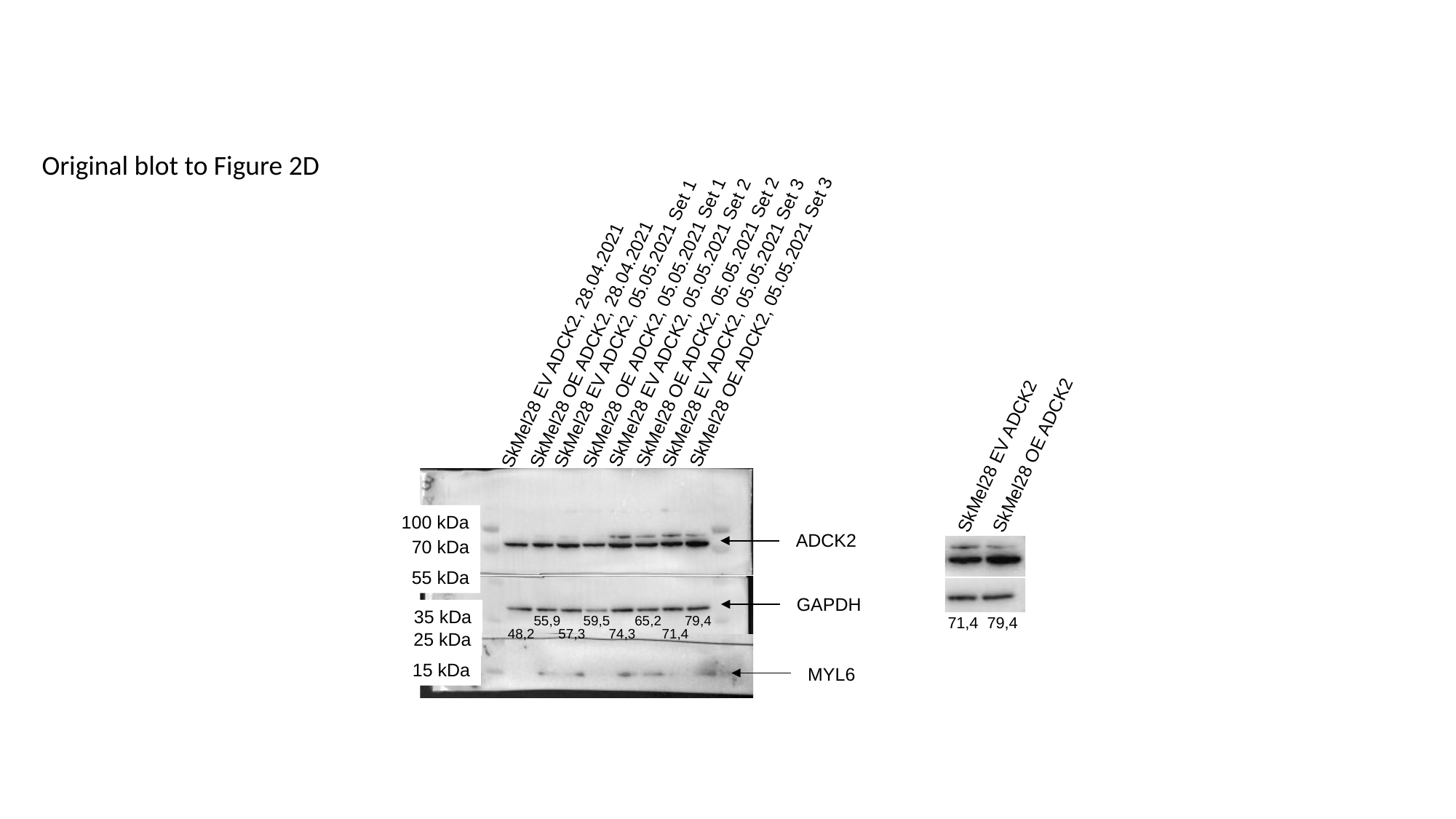

SkMel28 EV ADCK2, 05.05.2021 Set 3
SkMel28 EV ADCK2, 05.05.2021 Set 2
SkMel28 EV ADCK2, 05.05.2021 Set 1
SkMel28 OE ADCK2, 05.05.2021 Set 3
SkMel28 OE ADCK2, 05.05.2021 Set 2
SkMel28 OE ADCK2, 05.05.2021 Set 1
SkMel28 EV ADCK2, 28.04.2021
SkMel28 OE ADCK2, 28.04.2021
Original blot to Figure 2D
SkMel28 EV ADCK2
SkMel28 OE ADCK2
71,4
79,4
100 kDa
ADCK2
70 kDa
55 kDa
GAPDH
35 kDa
55,9
59,5
65,2
79,4
48,2
57,3
74,3
71,4
25 kDa
15 kDa
MYL6

## Slide 3
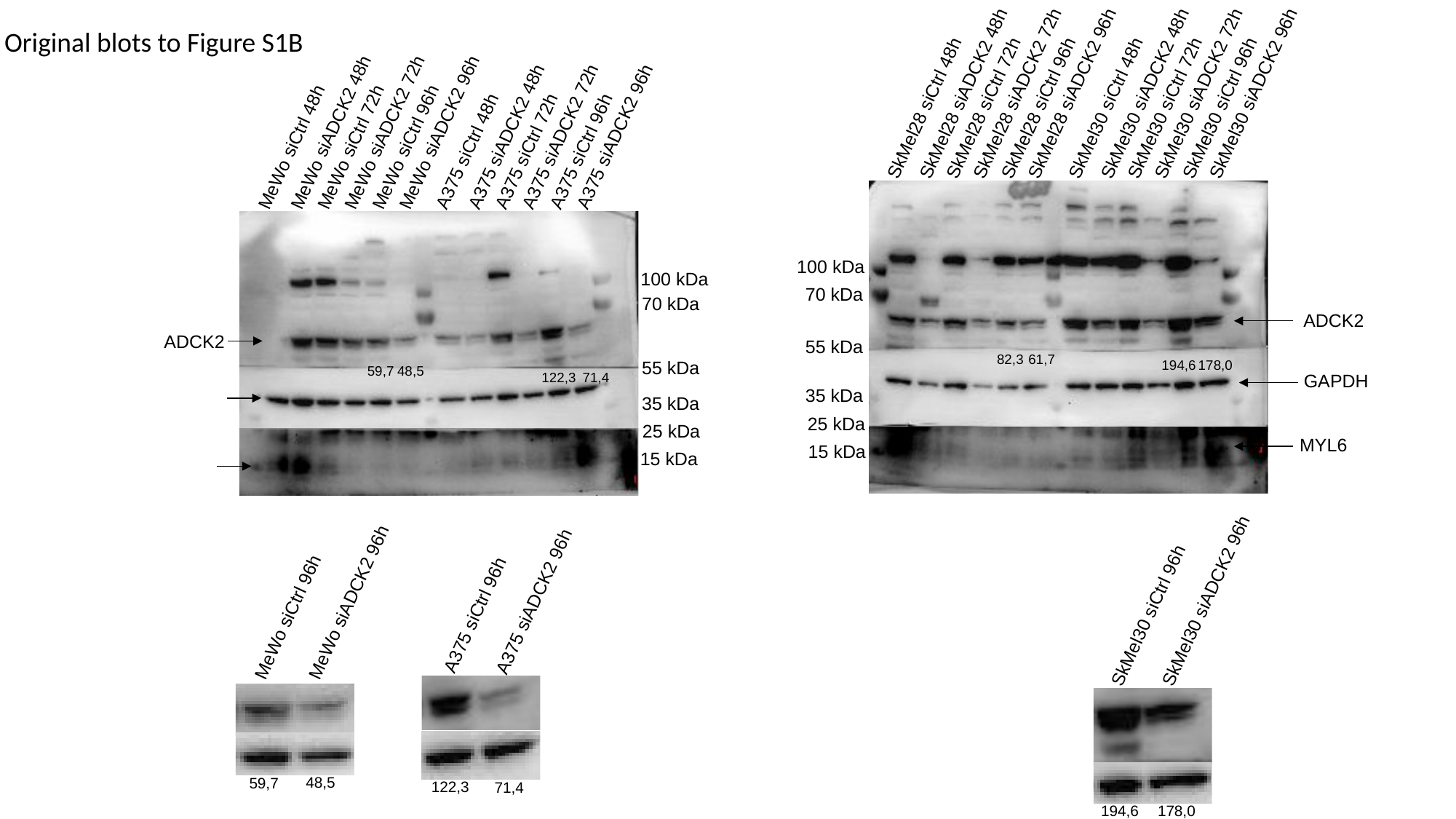

SkMel30 siADCK2 72h
SkMel30 siADCK2 96h
SkMel30 siADCK2 48h
SkMel30 siCtrl 48h
SkMel30 siCtrl 72h
SkMel30 siCtrl 96h
SkMel28 siADCK2 72h
SkMel28 siADCK2 96h
SkMel28 siADCK2 48h
SkMel28 siCtrl 48h
SkMel28 siCtrl 72h
SkMel28 siCtrl 96h
A375 siADCK2 72h
A375 siADCK2 96h
A375 siADCK2 48h
A375 siCtrl 48h
A375 siCtrl 72h
A375 siCtrl 96h
MeWo siADCK2 72h
MeWo siADCK2 96h
MeWo siADCK2 48h
MeWo siCtrl 48h
MeWo siCtrl 72h
MeWo siCtrl 96h
Original blots to Figure S1B
100 kDa
100 kDa
70 kDa
70 kDa
ADCK2
ADCK2
55 kDa
61,7
82,3
178,0
55 kDa
194,6
48,5
59,7
122,3
71,4
GAPDH
35 kDa
35 kDa
25 kDa
25 kDa
MYL6
15 kDa
15 kDa
A375 siADCK2 96h
A375 siCtrl 96h
122,3
71,4
MeWo siADCK2 96h
MeWo siCtrl 96h
48,5
59,7
SkMel30 siADCK2 96h
SkMel30 siCtrl 96h
194,6
178,0

## Slide 4
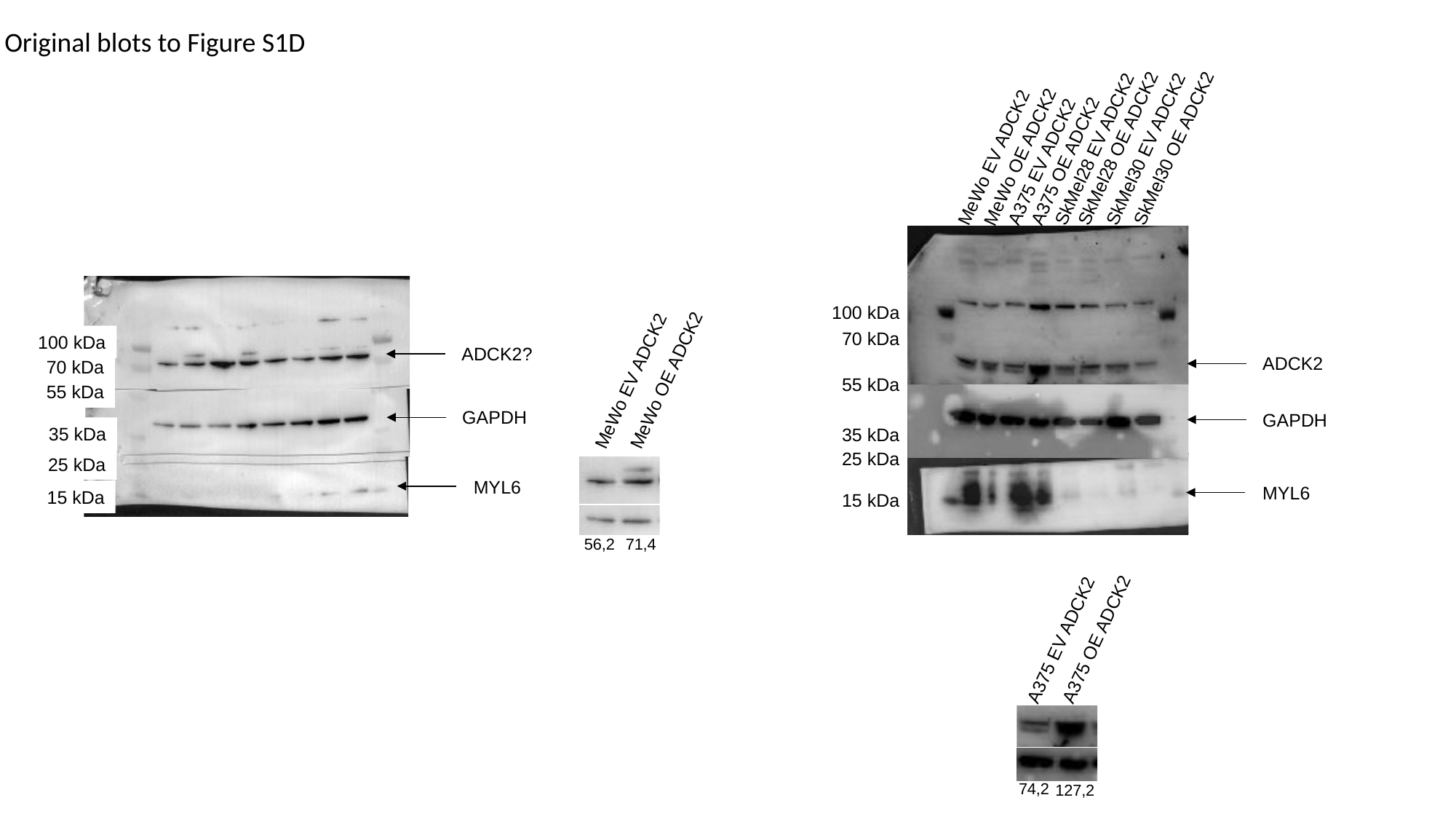

Original blots to Figure S1D
SkMel30 OE ADCK2
SkMel28 OE ADCK2
A375 EV ADCK2
SkMel28 EV ADCK2
SkMel30 EV ADCK2
MeWo OE ADCK2
A375 OE ADCK2
MeWo EV ADCK2
MeWo EV ADCK2
MeWo OE ADCK2
56,2
71,4
100 kDa
70 kDa
100 kDa
ADCK2?
ADCK2
70 kDa
55 kDa
55 kDa
GAPDH
GAPDH
35 kDa
35 kDa
25 kDa
25 kDa
MYL6
MYL6
15 kDa
15 kDa
A375 EV ADCK2
A375 OE ADCK2
74,2
127,2

## Slide 5
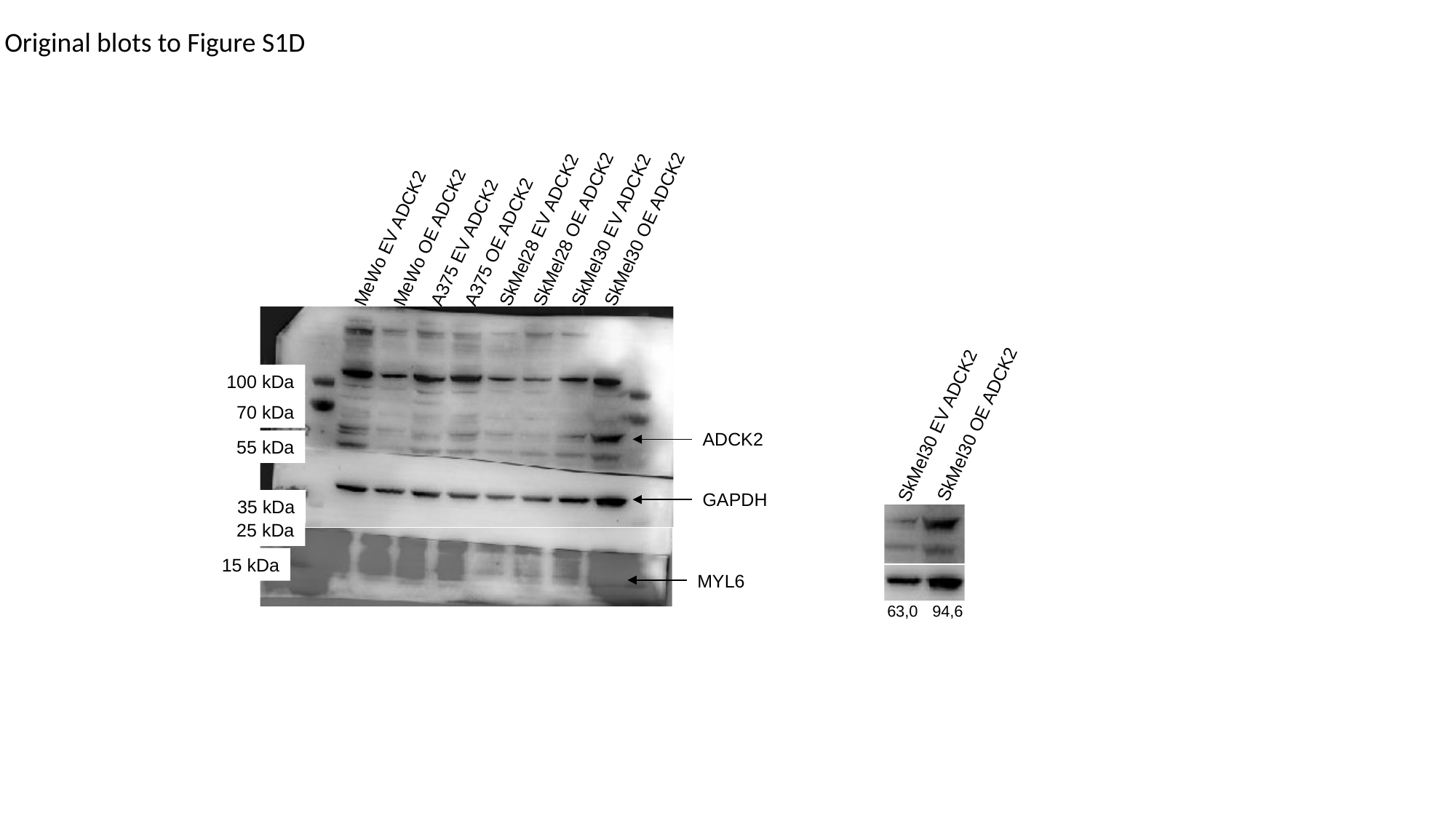

Original blots to Figure S1D
A375 EV ADCK2
SkMel28 EV ADCK2
SkMel30 EV ADCK2
MeWo OE ADCK2
A375 OE ADCK2
MeWo EV ADCK2
100 kDa
70 kDa
ADCK2
55 kDa
GAPDH
35 kDa
25 kDa
15 kDa
MYL6
SkMel30 OE ADCK2
SkMel28 OE ADCK2
SkMel30 OE ADCK2
SkMel30 EV ADCK2
94,6
63,0
