# Supplementary material for: ADCK2 Knockdown Affects the Migration of Melanoma Cells via MYL6
Source: Cancers (Basel). 2022 Feb 20;14(4):1071. doi: 10.3390/cancers14041071 (PMC8869929; doi:10.3390/cancers14041071)
Supplement: Supplementary file 1 [file cancers-14-01071-s001.zip › cancers-1546617-supplementary-send to xml.pdf]

cancers

MDPI

Supplementary Material

ADCK2 knockdown affects the migration of melanoma cells via MYL6

Marlene Vierthaler [1,2,3,4], Sun Qian[1,2,3,4], Yiman Wang[1,2,3,4], Tamara Steinfass[1,2,3,4], Juliane Poelchen[1,2,3,4], Thomas Hielscher[4,5], Daniel Novak[1,2,3] Viktor Umansky[1,2,3] and Jochen Utikal[1,2,3,*]

[1] Skin Cancer Unit, German Cancer Research Center (DKFZ), Heidelberg, Germany
[2] Department of Dermatology, Venereology and Allergology, University Medical Center Mannheim, Ruprecht Karl University of Heidelberg, Mannheim, Germany
[3] DKFZ-Hector Cancer Institute at the University Medical Centre Mannheim, Mannheim, Germany
[4] Faculty of Biosciences, Ruprecht Karl University, Mannheim, Germany
[5] Biostatistik, German Cancer Research Center (DKFZ), Heidelberg, Germany
* Correspondence: j.utikal@dkfz.de

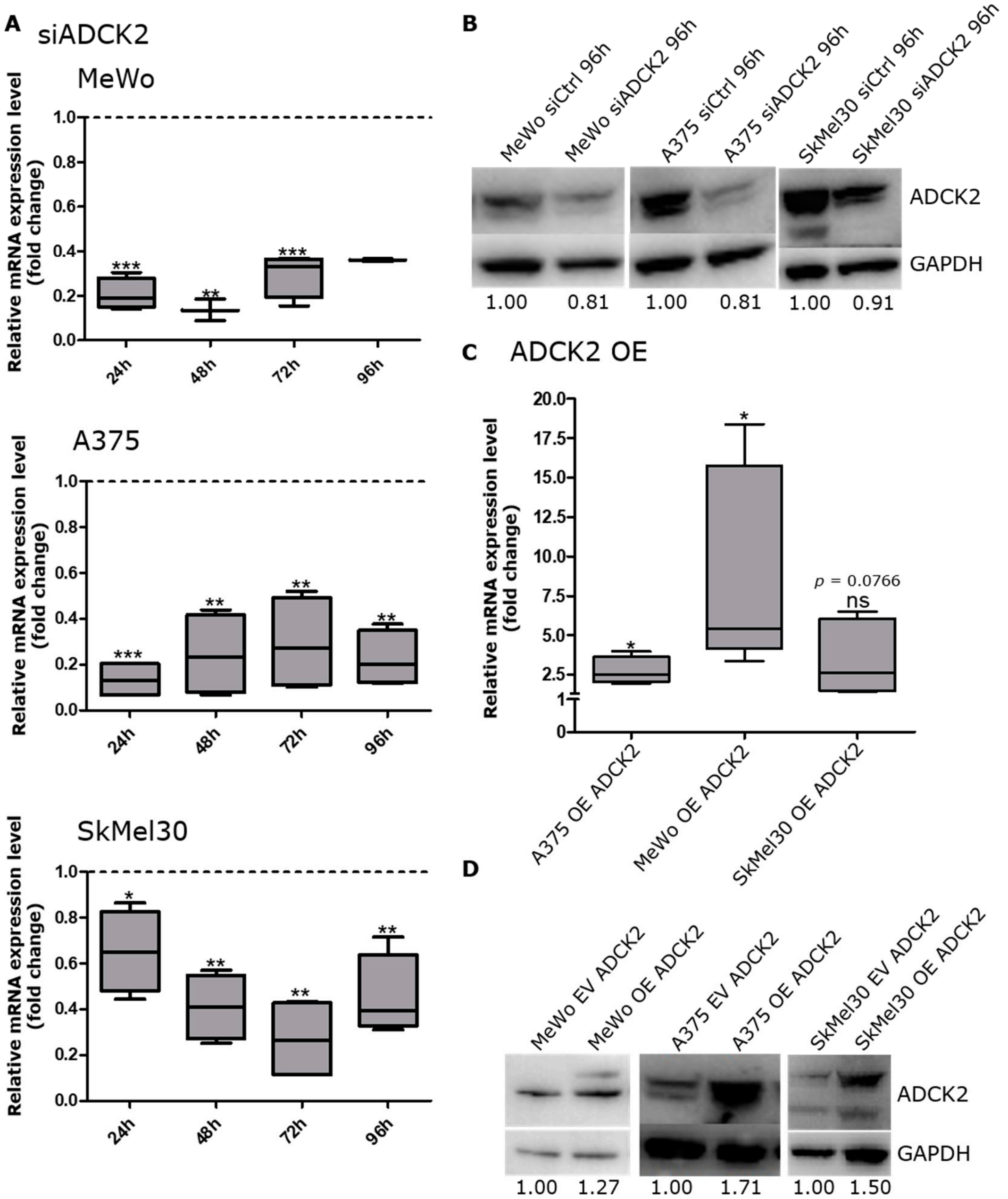

Figure S1. Validation of ADCK2 knockdown and overexpression. (**a**): ADCK2 knockdown validation. Treatment with siADCK2 significantly reduced mRNA expression in MeWo, A375 and SkMel30 cells as early as 24h and until 96h upon transfection. $n = 4$ (**b**): The ADCK2 knockdown was also validated via western blot analysis (representative images) (**c**): ADCK2 overexpression in MeWo, A375 and SkMel30 increased the mRNA expression level of ADCK2 by at least 2.5 fold compared to the empty vector control ($n \geq 4$). (**d**): Western blot analysis (representative images) confirmed the successful overexpression. All statistical analyses were done with paired t-test. *$p \leq 0.05$; ** $p \leq 0.01$; ***$p \leq 0.001$; ns = not significant.

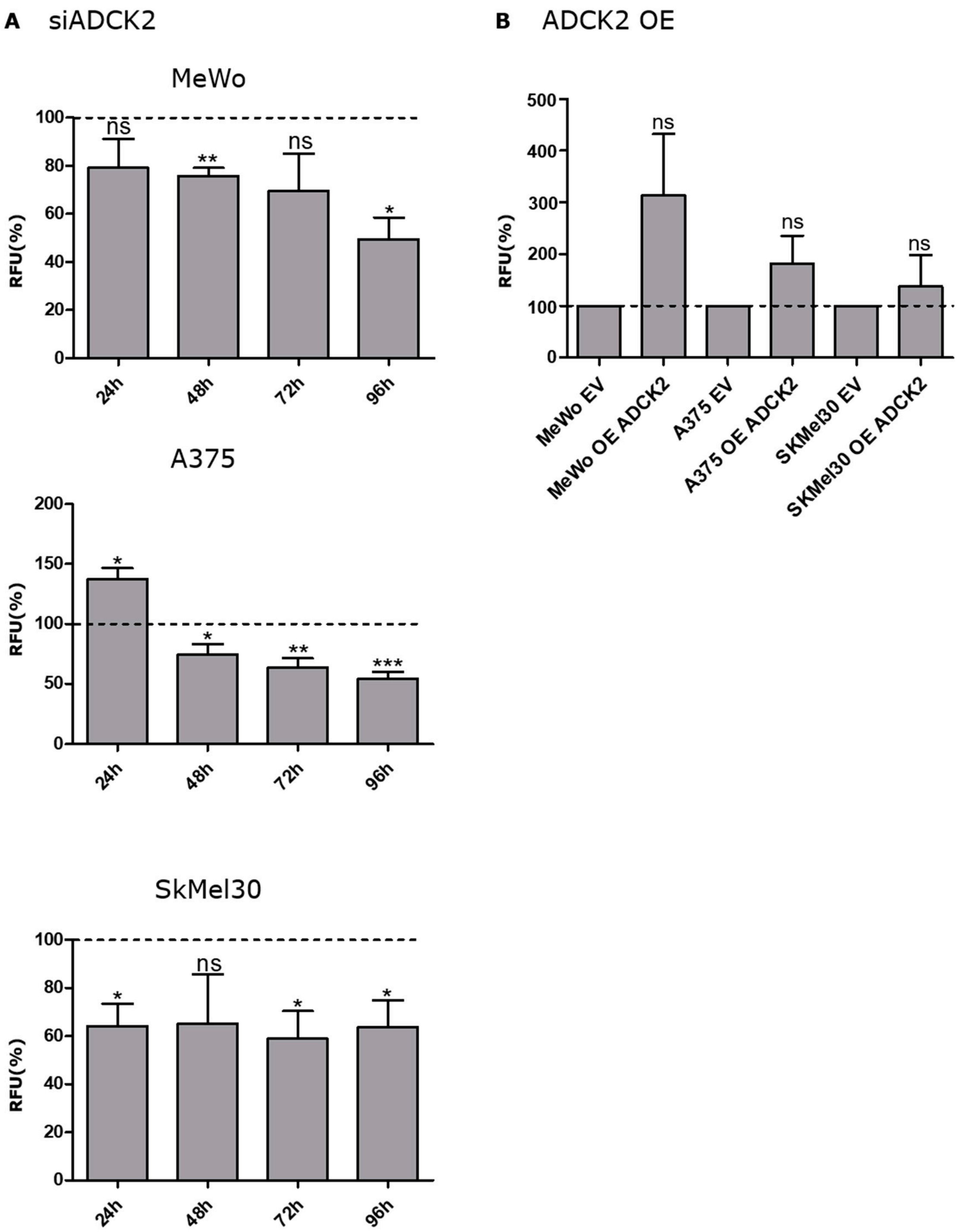

Figure S2. ADCK2 knockdown decreases cell viability of melanoma cells. (**a**): The cell viability of MeWo, A375 and SkMel30 decreased upon ADCK2 knockdown compared to control cells. This effect was strongest 96h upon transfection. $n = 6$ (**b**): Overexpression of ADCK2 did not alter the cell viability of MeWo, A375 or SkMel30 cells. $n = 5$ All statistical analyses were done with paired t-test. *$p \leq 0.05$; **$p \leq 0.01$; ***$p \leq 0.001$; ns = not significant. RFU = relative fluorescence units.

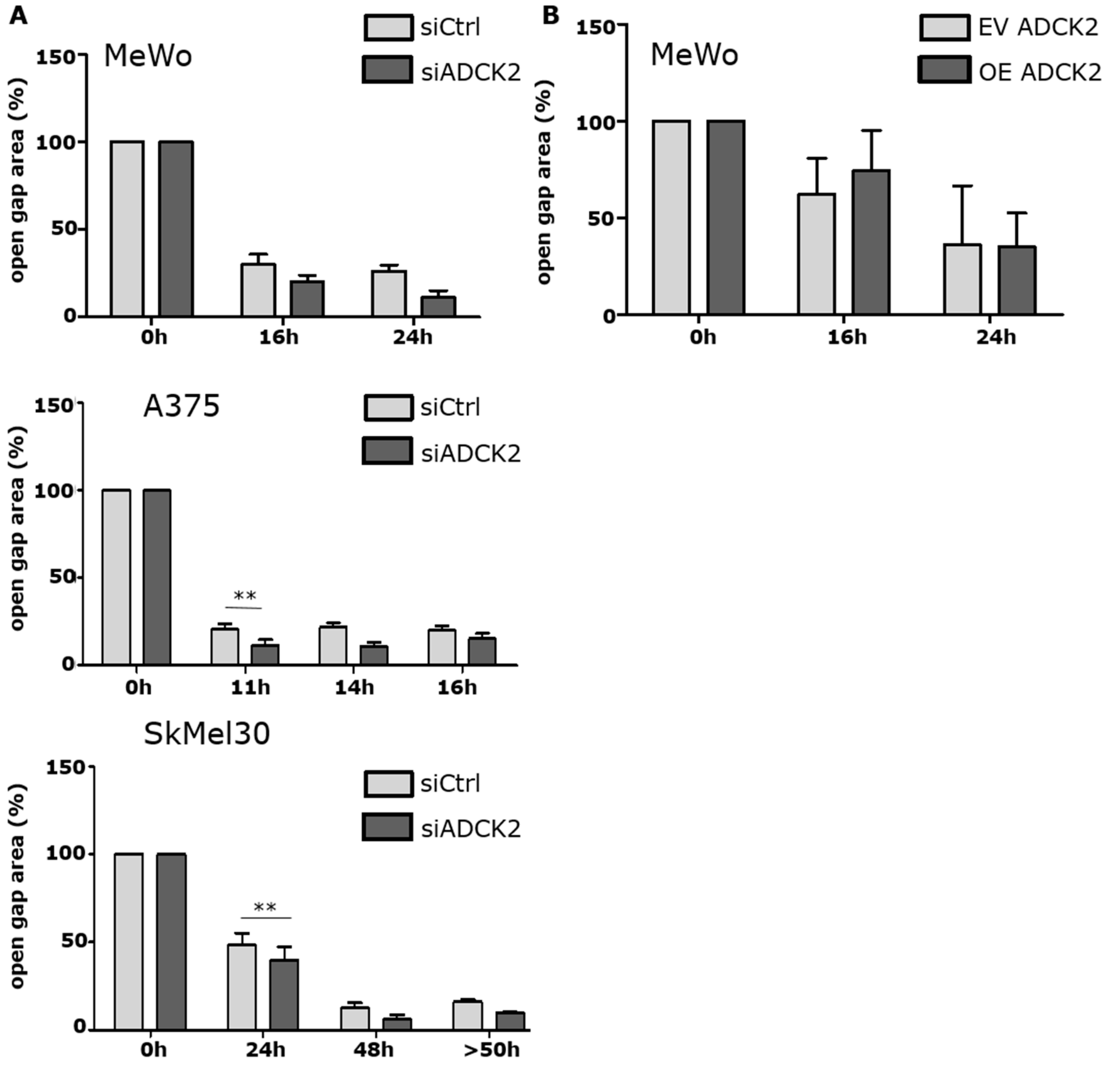

Figure S3. ADCK2 knockdown increases the migration capacity of melanoma cells. (**a**): Upon transfection with siADCK2 the migration capacity of A375 and SkMel30 was significantly higher. $n \geq 4$ (**b**): Overexpression of ADCK2 in MeWo cells resulted in a decreased migration capacity compared to empty vector control cells. $n = 6$ All statistical analyses were done with paired t-test. $^{**}p \leq 0.01$.

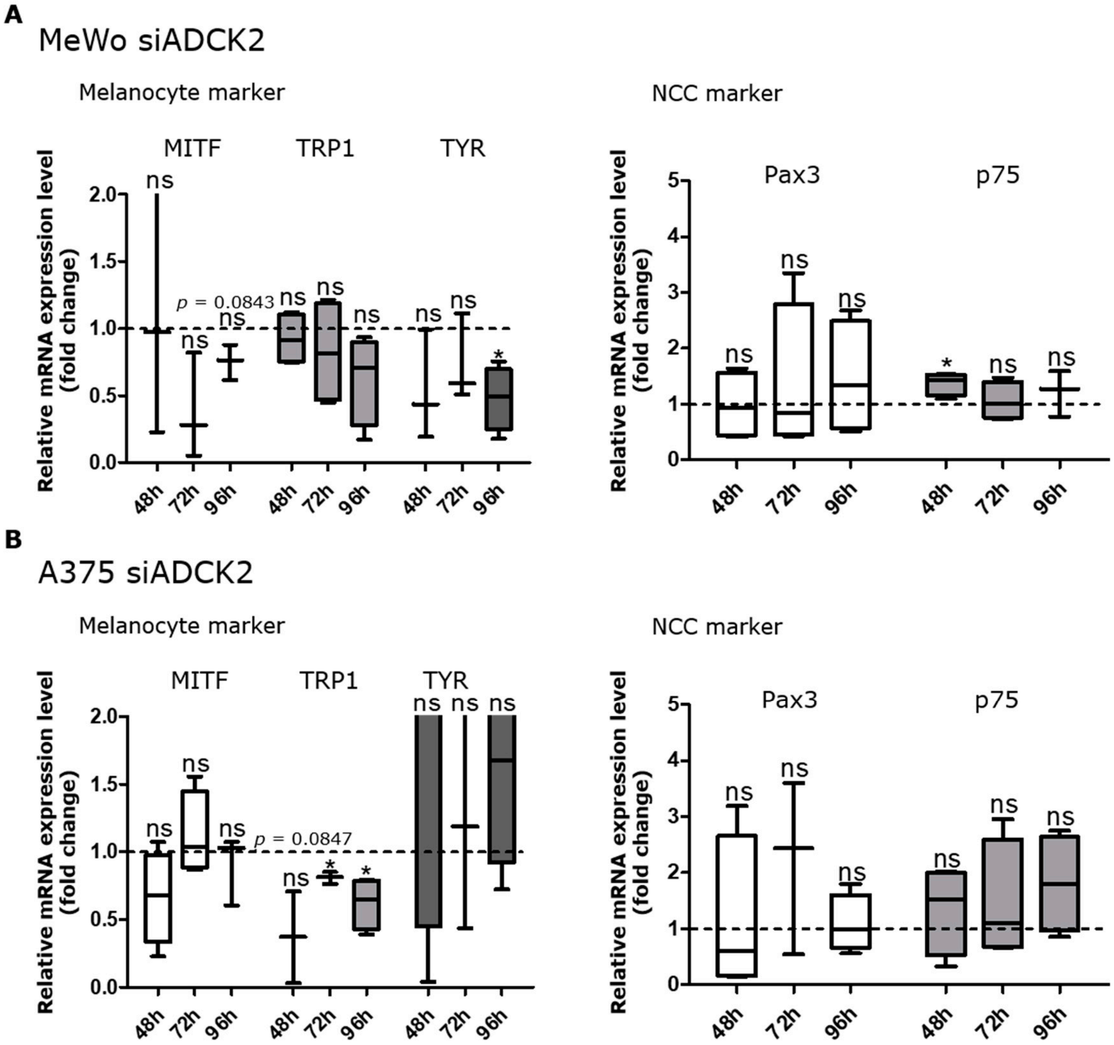

Figure S4. Melanoma cells adopted a more dedifferentiated phenotype after ADCK2 knockdown. (a): In MeWo cells the expression of the melanocyte marker TYR decreased significantly upon ADCK2 knockdown, while the expression level of the NC marker p75 was significantly higher . (b): A375 cells showed a significantly reduced expression of the melanocyte marker TRP1 upon ADCK2 knockdown. The expression of NC markers was unchanged compared to siControl transfected cells. $n = 4$ All statistical analyses were done with paired t test. *$p \leq 0.05$; ns = not significant.

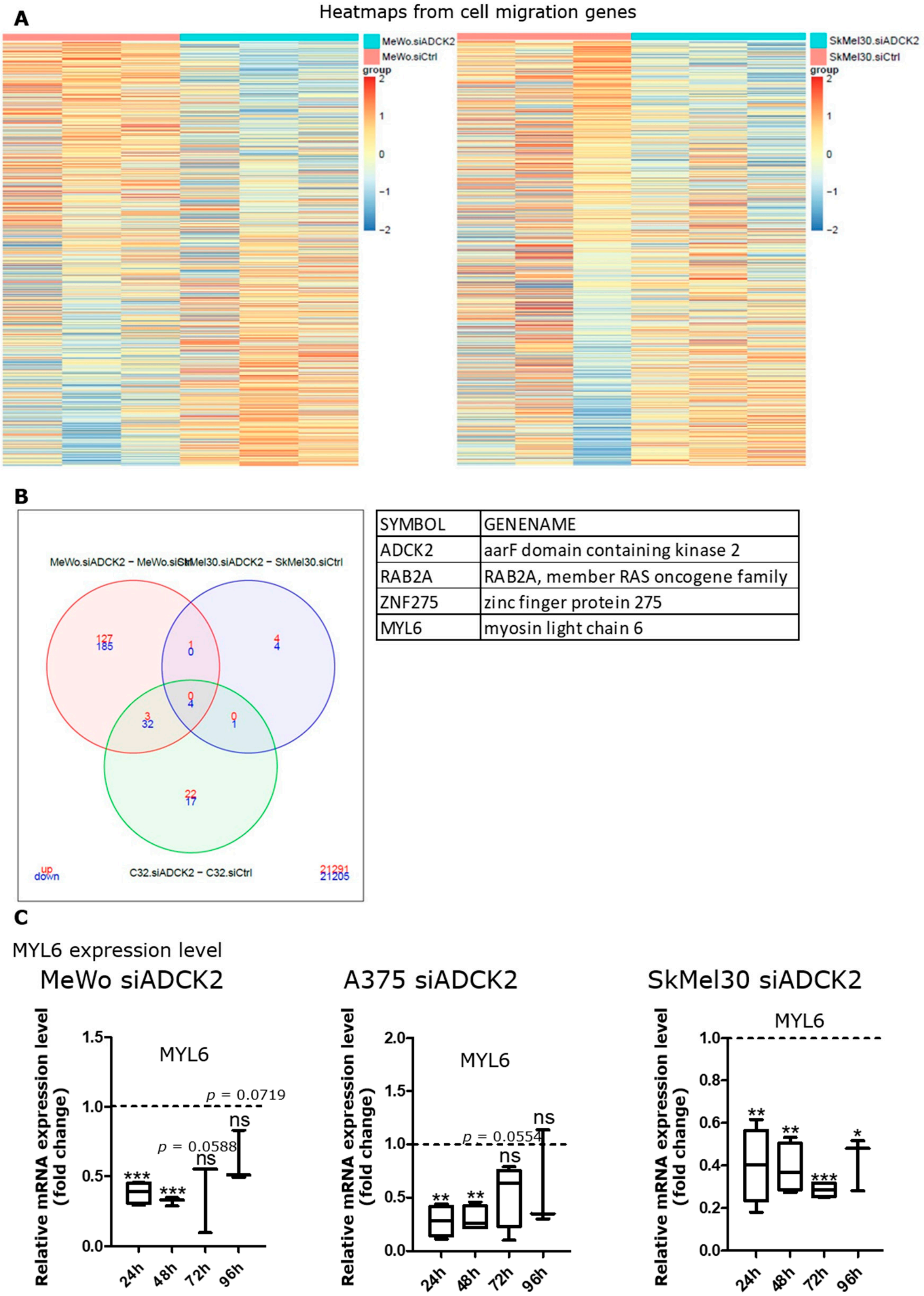

SYMBOL	GENENAME
ADCK2	aarF domain containing kinase 2
RAB2A	RAB2A, member RAS oncogene family
ZNF275	zinc finger protein 275
MYL6	myosin light chain 6

Figure S5. A gene expression array revealed MYL6 as a possible downstream factor of ADCK2. (**a**): Heatmaps showing the expression changes for all genes related to cell migration in MeWo (left side) and SkMel30 (right side) cells 24h upon ADCK2 knockdown and siControl tranfection. (**b**): Venn Diagram of three melanoma cell lines (MeWo, SkMel30, C32), all with siControl versus siADCK2 group. The four common downregulated genes in all cell lines upon ADCK2 knockdown are listed in the table next to the Venn diagram. (**c**): In MeWo, A375 and SkMel30 cells the knockdown of ADCK2 led to a lower mRNA expression level of MYL6 at all tested time points. $n = 4$ All statistical analyses were done with paired t-test. *$p \leq 0.05$; **$p \leq 0.01$; ***$p \leq 0.001$; ns = not significant.

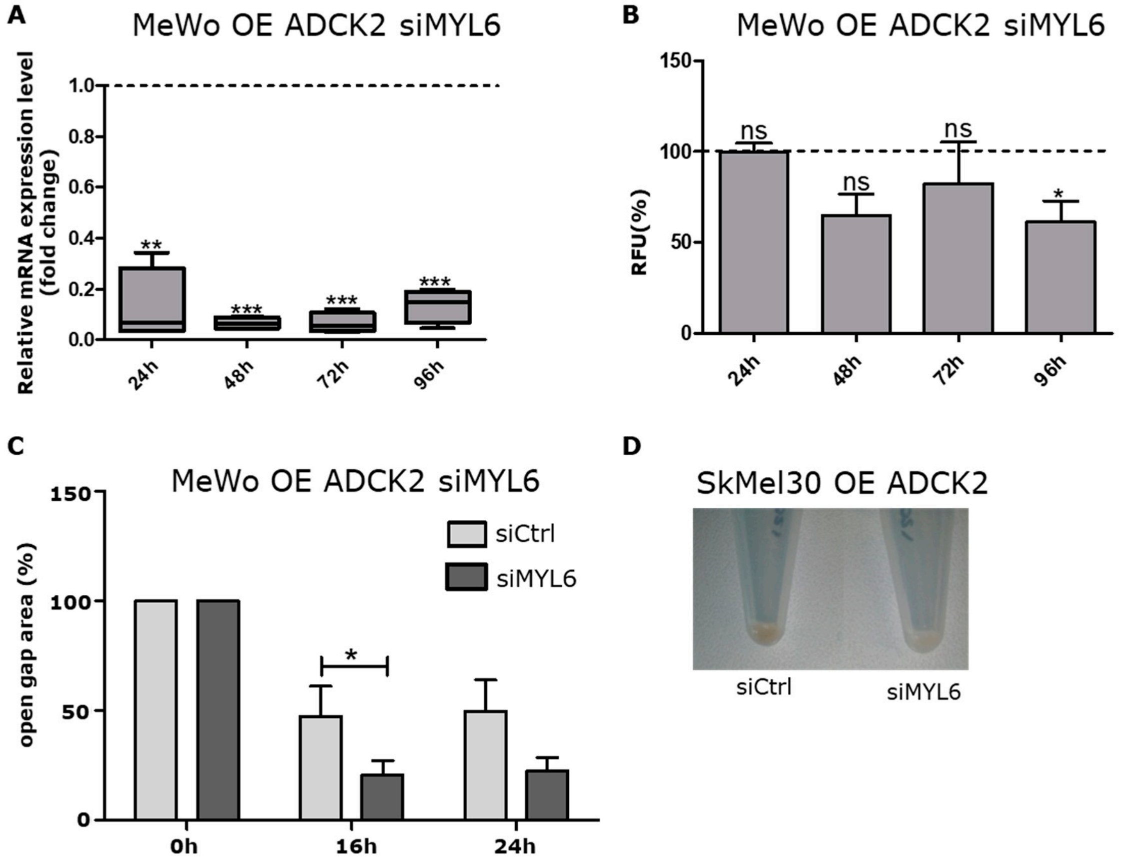

Figure S6. MYL6 knockdown can negate the effect of ADCK2 overexpression in melanoma cells. (**a**) In MeWo OE ADCK2 cells MYL6 mRNA expression levels were significantly downregulated 24, 48, 72 and 96h upon knockdown of MYL6 compared to siControl transfected cells (**b**): A knockdown of MYL6 in ADCK2 overexpressing MeWo cells led to a lower cell viability compared to control cells. $n = 6$ (**c**): A faster migration was seen for ADCK2 overexpressing cell lines transfected with siMYL6 compared to cells treated with siControl. $n = 5$ (**d**): Pellets of SkMel30 OE ADCK2 cells treated with siMYL6 were brighter compared to the control. All statistical analyses were done with paired t-test. *$p \leq 0.05$; **$p \leq 0.01$; ***$p \leq 0.001$; ns = not significant.

Table S1. Sequences of the primers used for qPCR.

	**Forward**	**Reverse**
ADCK2	TCT CGC AGA CCA GTC GGT TG	GCA ACA CTT TCA CTG CCA CG
Pax3	CTG TGC CCA GGA TGA TGC G	GAT CTT GTG GCG GAT GTG GT
p75	CGA CAA CCT CAT CCC TGT CT	GCT CTT CAA CCT CTT GAA GG
MITF	AGT GTT GAT AGT TTA TGG C	ACT TGG TGG GGT TTT CGA GG
TRP1	AGC AGT AGT TGG CGC TTT GT	TCA GTG AGG AGA GGC TGT TT
TYR	TTG TAC TGC CTG CTG TGG AG	CAG GAA CCT CTG CCT GAA AG
Sox5	CAG CCA GAG TTA GCA CAA TAG G	CTG TTG TTC CCG TCG GAG TT
FoxD3	CAT CCG CCA CAA CCT CTC	CAT ATG AGC GCC GTC TG
MYL6	GAA GAC CAG ACC GCA GAG TTC	TCC AGC ACC TTC ACA TTC ATC
RAB2A	CGT TCC ATC ACA AGG TCG TAT T	CTT AGC AGA CGT TTC CAT GAA GA
18S	GAGGATGAGGTGGAACGTGT	TCTTCAGTCGCTCCAGGTCT
